# Development and Validation of an MRI-Based Brain Volumetry Model Predicting Poor Psychomotor Outcomes in Preterm Neonates

**DOI:** 10.3390/jcm14061996

**Published:** 2025-03-15

**Authors:** Joonsik Park, Jungho Han, In Gyu Song, Ho Seon Eun, Min Soo Park, Beomseok Sohn, Jeong Eun Shin

**Affiliations:** 1 Department of Pediatrics, Yonsei University College of Medicine, Severance Children’s Hospital, 50-1 Yonsei-ro, Seodaemun-gu, Seoul 03722, Republic of Korea; parkjs87@yuhs.ac (J.P.); feagd@yuhs.ac (J.H.); igsong@yuhs.ac (I.G.S.); hseun@yuhs.ac (H.S.E.); minspark@yuhs.ac (M.S.P.); 2 Department of Radiology, Samsung Medical Center, Sungkyunkwan University School of Medicine, Seoul 03722, Republic of Korea

**Keywords:** FreeSurfer 1, preterm 2, neurodevelopment 3

## Abstract

**Background/Objectives**: Infant FreeSurfer was introduced to address robust quantification and segmentation in the infant brain. The purpose of this study is to develop a new model for predicting the long-term neurodevelopmental outcomes of very low birth weight preterm infants using automated volumetry extracted from term-equivalent age (TEA) brain MRIs, diffusion tensor imaging, and clinical information. **Methods**: Preterm infants hospitalized at Severance Children’s Hospital, born between January 2012 and December 2019, were consecutively enrolled. Inclusion criteria included infants with birth weights under 1500 g who underwent both TEA MRI and Bayley Scales of Infant and Toddler Development, Second Edition (BSID-II), assessments at 18–24 months of corrected age (CA). Brain volumetric information was derived from Infant FreeSurfer using 3D T1WI of TEA MRI. Mean and standard deviation of fractional anisotropy of posterior limb of internal capsules were measured. Demographic information and comorbidities were used as clinical information. Study cohorts were split into training and test sets with a 7:3 ratio. Random forest and logistic regression models were developed to predict low Psychomotor Development Index (PDI < 85) and low Mental Development Index (MDI < 85), respectively. Performance metrics, including the area under the receiver operating curve (AUROC), accuracy, sensitivity, precision, and F1 score, were evaluated in the test set. **Results**: A total of 150 patient data were analyzed. For predicting low PDI, the random forest classifier was employed. The AUROC values for models using clinical variables, MR volumetry, and both clinical variables and MR volumetry were 0.8435, 0.7281, and 0.9297, respectively. To predict low MDI, a logistic regression model was chosen. The AUROC values for models using clinical variables, MR volumetry, and both clinical variables and MR volumetry were 0.7483, 0.7052, and 0.7755, respectively. The model incorporating both clinical variables and MR volumetry exhibited the highest AUROC values for both PDI and MDI prediction. **Conclusions**: This study presents a promising new prediction model utilizing an automated volumetry algorithm to distinguish long-term psychomotor developmental outcomes in preterm infants. Further research and validation are required for its clinical application.

## 1. Introduction

Preterm births, occurring before 37 weeks of gestation, pose a high risk for neurodevelopmental impairments (NDI), necessitating post-discharge interventions to enhance outcomes [1,2,3]. Several global cohorts have reported NDI rates of 23% to 42% among children born at gestational ages between 22 and 25 weeks [4]. Notably, not all preterm infants experience developmental issues, but a subset faces significant motor and cognitive delays, requiring lifelong support.

Prior research has underscored the importance of term-equivalent age (TEA) magnetic resonance imaging (MRI) in predicting adverse neurodevelopmental outcomes [5,6]. Conventional MRI sequences are widely employed in clinical settings, and they frequently reveal white matter abnormalities, a primary pathology linked to long-term NDI [7,8]. Traditionally, the qualitative classification of white and gray matter injuries and quantitative assessments of periventricular white matter, corpus callosum, and gray matter gyrus maturation have been used as tools to predict these outcomes [9,10]. However, in cases where infants exhibit significant NDI without gross abnormalities in conventional MRI, there has been a pressing need to uncover additional diagnostic clues [11,12]. Therefore, diverse semi-quantitative methods using an MRI scoring system or manual measurement of brain growth in MRI plain image have been suggested and applied.

The previous study from the UK successfully reported calculated metrics using TEA diffusion MRI and combined clinical values to predict two-year language performance [12]. While the language scores remain meaningful, their implications within the first two years of life are limited, including the fact that 50% of language delays at 2 years spontaneously resolve over time [13,14]. In contrast, NDI in preterm infants is generally represented as motor or cognitive impairment, as well as language delay, which is typically confirmed within the first 2 years of life [15]. Moreover, the impairment of gross motor function suggests subsequent delays in other developmental domains, such as vision, hearing, language, and cognitive function. Moreover, the impairment of gross motor function suggests subsequent delays in other developmental domains, such as vision, hearing, language, and cognitive function [16,17]. Importantly, early intervention in the neurodevelopmental high-risk group improves prognosis for cognitive and motor development [18].

On the other hand, advancements in techniques, including volume measurements, diffusion tensor imaging (DTI), and functional MRI, have emerged to address the limitations of conventional MRI in understanding subsequent brain development. Recently, Valavani et al. suggested a prediction model for two-year language delay utilizing DTI with perinatal clinical information [19]. Selvanathan et al. reported that brain injury volume and location predict motor outcomes but not cognitive outcomes at 18 months of age, using manual segmentation of brain injury lesions followed by automated mapping [20].

Nevertheless, a definitive gold standard for predicting developmental outcomes still remains uncertain [21,22,23,24,25]. Moreover, there still remains a gap in translating imaging findings into actionable clinical interventions. Integrating advanced imaging techniques, such as automated volumetry and diffusion tensor imaging, with comprehensive clinical data offers a promising avenue for improving prediction accuracy and tailoring individualized care plans.

The purpose of this study is to develop a novel model for predicting the long-term neurodevelopmental outcomes of preterm infants. This model leverages automated volumetry extracted from term-equivalent age MRI, diffusion tensor imaging, and clinical information to enhance the accuracy of predictions.

## 2. Materials and Methods

### 2.1. Study Participants

This study was approved by the institutional review board of Severance Hospital (approval no., 4-2021-0329, approval date 4 May 2021). Preterm neonates hospitalized at Severance Children’s Hospital and born between January 2012 and December 2019 were consecutively included. The inclusion criteria encompassed infants with a birth weight under 1500 g who underwent both TEA MRI and assessments using the Bayley Scales of Infant and Toddler Development, Second Edition (BSID-II), at 18–24 months of corrected age (CA). Exclusion criteria comprised patients who had undergone ventriculoperitoneal shunt surgery due to intraventricular hemorrhage (IVH) and those with poor-quality MRI images. There were no sociodemographic health inequalities identified among the enrolled patients.

### 2.2. Neurodevelopmental Outcomes and Demographics

The Korean Bayley Scales of Infant and Toddler Development (K-BSID-II) were used for neurodevelopmental assessment at a CA 18–24 months. This assessment includes the psychomotor developmental index (PDI) for motor function and the mental developmental index (MDI) for cognitive function. The PDI is a score that measures a child’s motor development and is part of the Bayley Scales of Infant Development (BSID), and the MDI is a standardized score that measures an infant’s cognitive development. A diagnosis of NDI was defined as an MDI or PDI score of <85 (<−1 standard deviation [SD]), respectively. Clinical information was collected from medical records, including gestational age at birth, birth weight, sex, surgical necrotizing enterocolitis (NEC), grade 3–4 IVH, cystic periventricular leukomalacia (PVL), clinical seizures, retinopathy of prematurity, head circumference at discharge a diagnosis of cerebral palsy, and hearing impairment requiring assistive devices. The IVH classification was based on that of Papile et al. [26]. The clinical definitions for brain injury values (Grade 3–4 IVH and cystic PVL) were derived from the literature by Kidokoro et al. 2014 [12]. CA is the age a premature infant would be if they had been born on their due date and is calculated by subtracting the number of weeks or months the baby was born early from their actual age. TEA is defined as 37 to 44 weeks of gestational age for a preterm infant. All included patients underwent MRI at TEA following the standardized clinical protocol established at the institution.

### 2.3. MRI Protocols

A brain MRI was performed using a 3-T scanner (GE MRI 750 w, GE Healthcare, Milwaukee, WI, USA). The MRI examination included a 3D T1-weighted fast spoiled gradient-echo sequence and diffusion tensor imaging (DTI). (TR 9.84 s, TE 4.60 s, flip angle 8, FOV 16 cm, slice thickness 1 mm, slice spacing 1 mm for 3DT1WI) (TR 5643.2 s, TE 71 s, flip angle 90, FOV 16 m, slice thickness 2 mm, slice spacing 2 mm, b value = 1000, 32 directions for DTI). A fractional anisotropy (FA) map was created using the DTI source image on the MRI console.

### 2.4. Image Processing and Analysis

**Figure A1:**
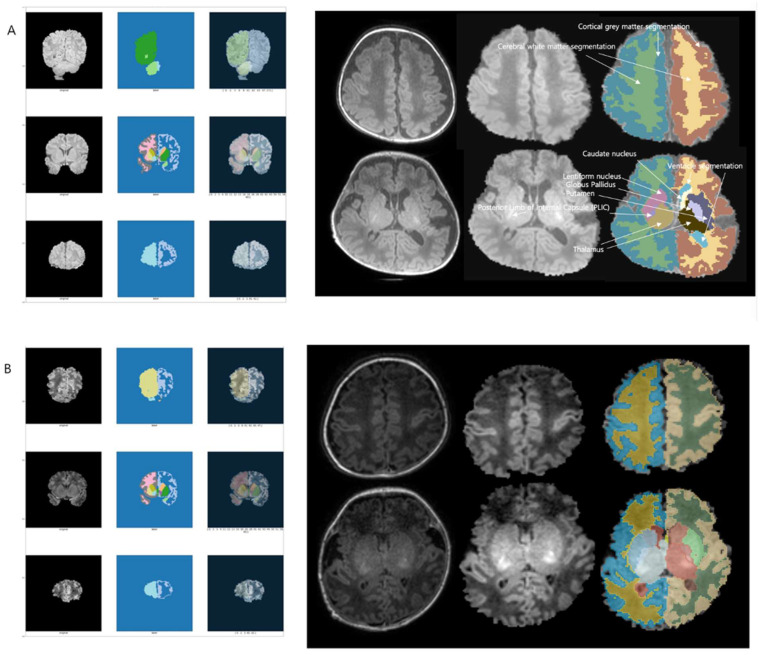
Example of Brain Segmentation Using Infant FreeSurfer Two representative examples of brain segmentation in infants using the Infant FreeSurfer pipeline. (**A**) illustrates a case with relatively successful segmentation. The first column shows the 3D T1-weighted MRI, the second column displays the segmentation mask, and the third column presents the overlay of the MRI and the segmentation mask. (**B**) depicts a less successful segmentation case. In this case, a significant portion of the brain cortex is missing, as it was erroneously removed during the skull stripping process.

The developmental outcome of the patient was blinded during the image processing and analysis. The 3D T1WI images were processed using Infant FreeSurfer 7.1.1. “https://surfer.nmr.mgh.harvard.edu/fswiki/infantFS”(accessed on 2 January 2025) (Figure A1). The volumetric data of the brain segmental volume, brain segmental volume without the ventricle, supratentorial volume, subcortical gray matter volume, right hemisphere cortex volume, left hemisphere cortex volume, total cortex volume, left hemisphere cerebral white matter volume, right hemisphere cerebral white matter volume, total cerebral white matter volume, mask volume, supratentorial volume without ventricle voxel, brain segmental volume without ventricle surface, and ventricle choroid plexus volume, were derived from Infant FreeSurfer. The segmentation and image processing pipeline has been described elsewhere [27]. The mean and standard deviation of the FA values were derived from the bilateral posterior limb of the internal capsule (PLIC), with manual segmentation by a neuroradiologist who is blind to the result. All the mentioned data were utilized as MR volumetry variables.

### 2.5. Machine Learning and Statistical Analysis

Two model groups were developed, one for PDI prediction and the other for MDI prediction. A stratified random split divided the enrolled patients into a 70:30 ratio, with the stratification factor being the PDI score. After preparing the training set, three models were developed to predict a low PDI. Model 1 used only MR volumetry data, Model 2 used clinical features exclusively, and Model 3 was developed using both MR volumetry and clinical features. These models were created using the random forest classifier and logistic regression methods. The clinical variables used were gestational age at birth, birth weight, sex, necrotizing enterocolitis, grade 3–4 IVH, cystic PVL, seizures, and retinopathy of prematurity. Hyperparameter tuning was conducted using 3-fold cross-validation during training, with a grid search employed to optimize the parameters. After training, the classifier that showed a higher area under the receiver operating curve (AUROC) on 3-fold cross-validation was applied to the test set. The AUROC and its 95% confidence interval (CI), area under the precision–recall curve (AUPRC), accuracy, sensitivity, precision, and F1 score were evaluated using the test set. The same process was performed for the MDI prediction. All processes up to this point were carried out using Python 3 (Python Software Foundation, Wilmington, DE, USA) with the Scikitlearn library v0.21.2 and R software (version 3.5.1; R Foundation for Statistical Computing, Vienna, Austria). Statistical significance was set at *p* < 0.05; *p*-values were two-sided.

## 3. Results

### 3.1. Patient Demographics

**Figure 1:**
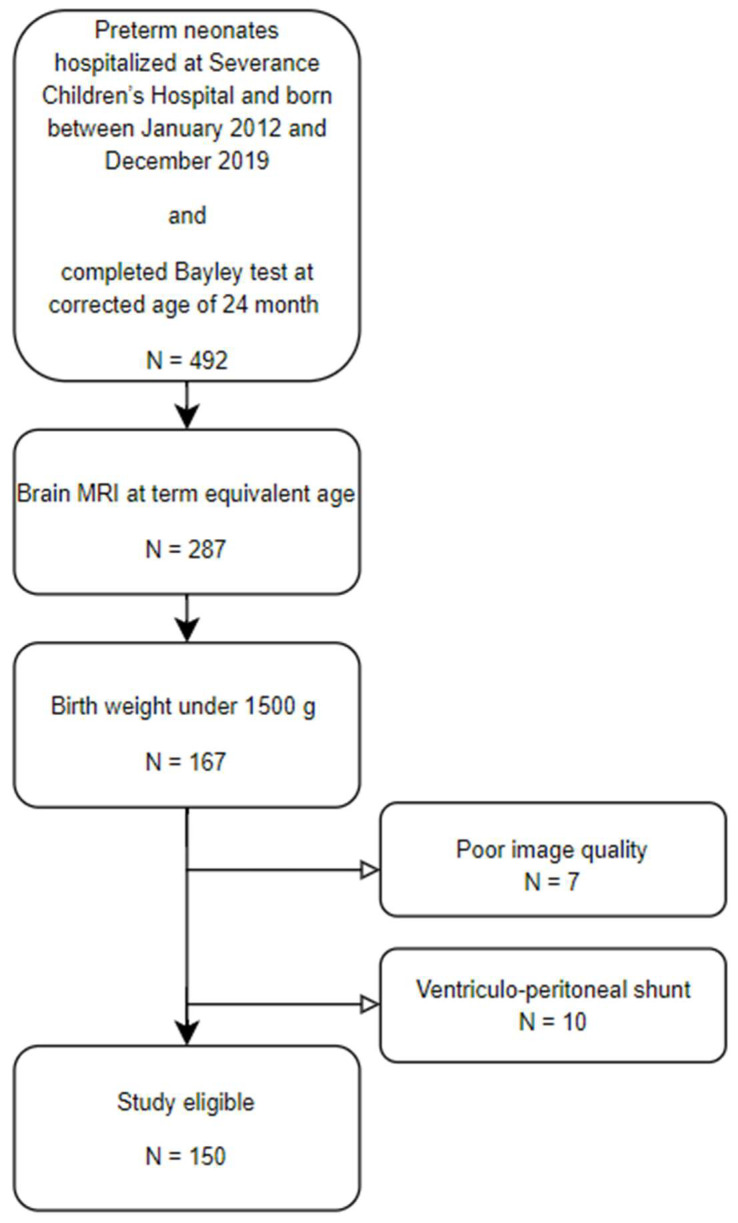
Patient selection flow chart.

A total of 167 Very Low Birth Weight Infants (VLBWIs) successfully completed both TEA MRI and the Bayley Scale at 18–24 months of CA. Ten patients were excluded owing to having a ventriculoperitoneal shunt, and another seven patients were excluded because of poor-quality MRI images (Figure 1). Finally, data from 150 patients were analyzed. The baseline characteristics of the enrolled patients are presented in Table 1. The median gestational age of the patients was 28.7 weeks, and the median birth weight was 1005 g. Among these patients, 78 (52.0%) were male. The proportion of patients with neurologic complications was 4.7%, with 3–4 IVH, 7.3% with cystic PVL, and 4.7% with clinical seizures. Forty-six (30.7%) patients had a head circumference below 10 percentiles at discharge.

At a corrected age of 18–24 months, 77 (51.3%) patients were diagnosed with mental developmental delay, 49 (32.7%) had motor developmental delay, and 42 (28.0%) had both. Hearing loss requiring a hearing aid was diagnosed in 2.0% of the patients, and cerebral palsy was diagnosed in 7.3% of the population (Table 2).

### 3.2. Prediction Model Performances

**Figure 2:**
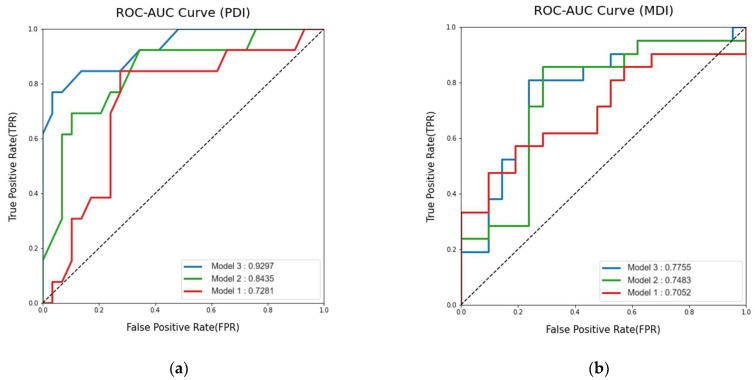
Model 1 used only MR volumetry data, Model 2 used clinical features exclusively, and Model 3 was developed using both MR volumetry and clinical features: (**a**) Area Under the Curve of the Receiver Operating Characteristic for predicting low performance developmental index (PDI); (**b**) Area Under the Curve of the Receiver Operating Characteristic for predicting low mental developmental index (MDI).

For low PDI prediction, the AUROC of random forest models using clinical variables, MR volumetry, and both clinical variables and MR volumetry were 0.84 (95% CI: 0.85–1.00), 0.73 (95% CI: 0.56–0.90), and 0.93 (95% CI: 0.71–0.98), respectively (Figure 2). The AUPRC were 0.51 (95% CI: 0.30–0.74), 0.41 (95% CI: 0.19–0.45), and 0.68 (95% CI: 0.47–0.87), respectively. The AUROC of logistic regression models using clinical variables, MR volumetry, and both clinical variables and MR volumetry were 0.67 (95% CI: 0.47–0.86), 0.82 (95% CI: 0.67–0.96), and 0.82 (95% CI: 0.67–0.97), respectively. The AUPRC were 0.42 (95% CI: 0.23–0.65), 0.31 (95% CI: 0.22–0.64), and 0.54 (95% CI: 0.33–0.74), respectively.

For low MDI prediction, the AUROC of the logistic regression model using clinical variables, MR volumetry, and both clinical variables and MR volumetry were 0.75 (95% CI: 0.63–0.92), 0.71 (95% CI: 0.54–0.87), and 0.78 (95% CI: 0.59–0.91), respectively (Figure 2). The AUPRC were 0.57 (95% CI: 0.39–0.74), 0.67 (95% CI: 0.49–0.84) and 0.62 (95% CI: 0.45–0.79), respectively, The AUROC of random forest models using clinical variables, MR volumetry, and both clinical variables and MR volumetry were 0.71 (95% CI: 0.55–0.87), 0.75 (95% CI: 0.60–0.90) and 0.76 (95% CI: 0.61–0.91), respectively. The AUPRC were 0.63 (95% CI: 0.46–0.80), 0.58 (95% CI: 0.41–0.76), and 0.67 (95% CI: 0.49–0.85), respectively.

Model 3, using both clinical variables and MR volumetry, showed the highest AUROC for both PDI and MDI predictions. The random forest model showed a higher AUROC for PDI prediction than the logistic regression model. The accuracy, sensitivity, precision, and F1 score of this random forest PDI prediction model were 0.86, 0.84, 1, and 0.7, respectively. For MDI prediction, the logistic regression model showed a higher AUROC than the random forest model. The accuracy, sensitivity, precision, and F1 score of this logistic regression MDI prediction model were 0.69, 0.81, 0.65, and 0.72, respectively (Table A1).

The random forest model for PDI prediction utilized seizure, IVH, cystic PVL, birth weight, SD of FA from the right PLIC, mean FA from the left PLIC, subcortical gray matter volume, left hemisphere cortical volume, right hemisphere cortical volume, total cortical volume, total gray matter volume, left cerebral white matter volume, right cerebral white matter volume, total brain volume, supratentorium without ventricle volume, brain without ventricle volume, and ventricle volume. For MDI prediction, the logistic regression model included VP shunt status, IVH, cystic PVL, NEC operation history, head circumference (HC) < 10p at discharge, sex, birth weight, mean FA from the right PLIC, left cerebral white matter volume, and right cerebral white matter volume as variables.

## 4. Discussion

In this study, we aimed to investigate whether MRI volumetry and DTI at TEA in combination with clinical information can predict neurodevelopmental outcomes in preterm infants at a corrected age of 18–24 months. The random forest classifier model using both clinical variables and MR information had the highest AUROC for predicting abnormal motor function compared with clinical or MR-only models. To predict abnormal cognitive function, the logistic regression model using both clinical variables and MR volumetry had the highest function; however, it showed a lower AUROC than the motor function prediction model.

MRI has long been regarded as a powerful tool for predicting the long-term neurodevelopmental morbidities of preterm infants [28]. In the current era, neonatologists often rely on information obtained from TEA MRI to predict long-term developmental outcomes by recognizing well-known patterns, such as gyral maturation of gray matter or white matter lesions [29,30]. Traditionally, these interpretations have been performed by expert neuroradiologists and neonatologists who combine clinical information to provide subjective explanations in real-world clinical practice.

Recent studies have designed various traditional binary statistical models that incorporate clinical data and MRI to predict morbidities in preterm and full-term perinatally asphyxiated infants [30,31]. Additionally, MRI volumetrics in preterm infants have been discussed in previous articles to improve predictive capabilities [32,33,34]. A previous study by Shin et al. developed a model with 10 relevant features extracted from T1- and T2-weighted MRI images, showing an AUROC value of 0.902 in predicting PDI performance, with the white matter volume of the posterior limb of the internal capsule being the best parameter among image features [35]. However, this study had limitations in handling raw image data segmentation by human experts.

A meta-analysis by Romberg et al. explored 13 articles discussing volumetric data in TEA MRIs of preterm infants [36]. Among these, ten studies employed manual or semiautomatic segmentation, with only three studies purely relying on automated segmentation [37,38,39]. Vasu et al. correlated volumetric data with nutritional clinical information but did not focus on explaining long-term outcomes [39]. Moeskops et al. concentrated on the automation of cortical morphology, while Kamino et al. failed to demonstrate a correlation between white matter volumetrics and long-term neurodevelopmental outcomes [37,38].

In our comparison of the clinical model, the MRI volumetry model, and the combined model, the combined model consistently demonstrated superior AUROC in predicting MDI across both logistic regression and random forest approaches. This underscores the significant enhancement in predictive accuracy achieved by integrating MRI volumetry data with clinical factors. However, for PDI prediction, the logistic regression model showed no AUROC improvement with the combined model compared to the MRI volumetry model alone, possibly reflecting the small size of our test set and the absence of significant findings. In contrast, the random forest model revealed an increased AUROC for the combined model over the volumetry model in PDI prediction.

Furthermore, the predictive accuracy for PDI was generally higher than for MDI, suggesting that volumetric information plays a more critical role in predicting motor function decline. This could indicate that brain volume metrics are more predictive of motor function outcomes, which aligns with previous clinical beliefs with conventional MRI [5,40]. A recent study has also suggested that predicting cognitive function based on neonatal information is less accurate compared to predicting motor function; maternal education has been identified as a stronger predictor of cognitive function than MRI data [20].

Our model exhibits strength in its fine prediction performance and using an automated segmentation volumetric model for neonatal age based on raw images. The previous study by Valavani et al. achieved impressive accuracy metrics, with an accuracy of 91%, a sensitivity of 86%, and a specificity of 96% [19]. Moving forward, we could explore the integration of additional image refinement technologies, such as “skeletonization,” mentioned in the aforementioned study, to further enhance predictions in developmental domains beyond motor skills. Nevertheless, as our model prioritizes high sensitivity to minimize missed cases, this may reduce specificity. Since the AUROC analysis inherently reflects this trade-off, different threshold settings can be adjusted to optimize specificity based on clinical needs.

Accurately predicting the future neurodevelopmental outcomes of infants discharged from the neonatal intensive care unit is not only a concern for parents but also holds significant clinical importance. It allows for the early screening of patients who may benefit from interventions, ultimately improving long-term outcomes. While neonatologists routinely obtain conventional MRI data from their preterm patients, the information has been limited until now. Our model opens up new possibilities for providing more automated information without the need for additional human resources in the real-world clinical setting. In an effort to explore the clinical utility of the model, we provided the imaging data, imaging interpretation reports from radiologists, and clinical information of the patients included in the study to two independent neonatologists who were blinded to the study. They were asked to predict low MDI and PDI scores using the same cutoff as the study (<85) at a corrected age of 18–24 months. Although the differences in clinicians’ personal experience and attitudes toward prognosis should be taken into account, the prediction of low MDI scores was successful in 40.7% and 32.9% of cases, respectively, while the prediction of low PDI scores was successful in 43.4% and 58.7% of cases, respectively. These results are lower compared to the performance of the model in our study. Therefore, we cautiously anticipate that our model could at least assist clinicians in making their judgments. With further refinement of this model and the development of a user-friendly software system that seamlessly integrates into existing workflows, it has the potential to serve as a valuable clinical decision support tool. Moreover, collecting more standardized and quantitative data through MRI could serve as a valuable resource for future preterm brain MRI research. This approach would be particularly beneficial in resource-limited settings, especially those without access to pediatric neuroradiologists, as it would enable standardized outcomes and provide additional resources to support clinical practice at peripheral sites.

We acknowledge several limitations in our study. First, our data collection was limited to a single center, and we conducted only internal validation. Future studies should leverage multi-center prospective cohorts to develop and validate robust prediction models, incorporating large sample sizes and independent datasets. Second, our study solely employed Infant FreeSurfer for brain MRI volume segmentation. It is important to note that the warping process used in this segmentation approach may not be optimal for cases where the subject’s brain deviates significantly from normal anatomy, such as in the examples presented in Figure A1. Extreme variations in brain shape may affect the accuracy of the resulting segmentation. In addition, the segmentation quality was assessed through visual inspection of the MRI scans prior to analysis. The reliance on qualitative inspection is a limitation of the current approach. Stronger segmentation tools for newborns are continuously evolving, and future research may benefit from exploring alternative algorithms beyond Infant FreeSurfer [41]. Third, in our methodology, FA maps were generated in real-time directly by the scanner console. While this approach enhances clinical applicability by eliminating the need for additional post-processing, it is important to acknowledge that these console-generated FA maps may exhibit increased sensitivity to artifacts and distortion compared to those produced after motion correction on a dedicated DTI workstation. Notably, regions such as the posterior limb of the internal capsule are generally less prone to these artifacts, but the overall susceptibility remains a limitation of our technique. In addition, for more robust and consistent predictions, utilizing a DTI atlas for measuring FA is recommended over manual techniques. Future studies should consider this approach for improved accuracy and reliability. Additionally, while MRI volumetry data are a valuable tool, our analysis lacks the evaluation of developmental influencing factors that are not readily apparent in conventional imaging. Specifically, functional aspects, such as connectivity, which may play a critical role in neurodevelopment, were not considered in this study. Lastly, we can integrate image refinement techniques to enhance our predictions, as demonstrated in previous studies [19].

## 5. Conclusions

In conclusion, our study presents a novel prediction model employing an automated volumetry algorithm that significantly differentiates long-term psychomotor developmental outcomes in preterm infants. This model complements conventional MRI, offering the potential to enhance clinical practice and provide valuable information for better patient care and intervention strategies.

## Figures and Tables

**Table 1:** Baseline characteristics of the patients at NICU discharge.

Clinical Variables (*n* = 150)	Median [IQR] or *n* (%)
Gestational weeks at birth	28.7 [26.8; 30.1]
Birth weight	1005.0 [832.5; 1275.0]
Male sex	78 (52.0%)
Inborn	138 (92.0%)
Intraventricular hemorrhage grade 3 and 4	7 (4.7%)
Cystic periventricular leukomalacia	11 (7.3%)
Clinical seizure	7 (4.7%)
Retinopathy of prematurity	27 (18.0%)
Surgical necrotizing enterocolitis	3 (2.0%)
Head circumference below 10th percentile at discharge	46 (30.7%)

*Continuous variables are represented as median [IQR]. Categorical variables are represented as number (%). Abbreviations: NICU, neonatal intensive care unit, IQR, interquartile range.*

**Table 2:** Neurodevelopmental outcomes at 18–24 months of corrected age.

Developmental Outcomes (*n* = 150)	*n* (%)
MDI < 85	77 (51.3%)
PDI < 85	49 (32.7%)
MDI and PDI, both < 85	42 (28.0%)
Any one of MDI or PDI < 85	84 (56.0%)
Deafness requiring hearing aids	3 (2.0%)
Cerebral palsy at 2 years old	11 (7.3%)

Data are represented as number (%). Abbreviations: MDI, mental developmental index; PDI, performance developmental index.

## Data Availability

The datasets generated and/or analyzed during the current study are not publicly available due to patient confidentiality rights but are available from the corresponding author upon reasonable request.

## Abbreviations

The following abbreviations are used in this manuscript:

NDI	neurodevelopmental impairments
TEA	term-equivalent age
MRI	magnetic resonance imaging
DTI	diffusion tensor imaging
BSID-II	Bayley Scales of Infant and Toddler Development, Second Edition
K-BSID-II	Korean-Bayley Scales of Infant and Toddler Development
PDI	psychomotor developmental index
MDI	mental developmental index
PVL	periventricular leukomalacia
IVH	intraventricular hemorrhage
CA	corrected age
FA	fractional anisotropy
PLIC	posterior limb of the internal capsule
AUROC	area under the receiver operating curve
CI	confidence interval
IQR	interquartile range
VLBWIs	very low birth weight infants
FA	fractional anisotropy
PLIC	posterior limb of the internal capsule
NEC	necrotizing enterocolitis
HC	head circumference
AUPRC	area under the precision recall curve
SD	standard deviation
NICU	neonatal intensive care unit
ELBWIs	extremely low birth weight infants

## Appendix A

**Table A1:** Predictive score values from each MR volumetry, clinical features, and combined both MR volumetry and clinical features.

	AUROC (95% Confidence Interval)	Accuracy	Sensitivity	Precision	F1 Score
**PDI predictor**					
Model 1	0.73 (0.56–0.90)	69.0	53.8	50.0	0.52
Model 2	0.84 (0.71–0.98)	83.8	84.6	55.0	0.67
Model 3	0.93 (0.85–1.00)	85.7	53.8	100.0	0.70
**MDI predictor**					
Model 1	0.71 (0.54–0.62)	61.9	71.4	60.0	0.65
Model 2	0.75 (0.59–0.91)	73.8	76.2	72.7	0.74
Model 3	0.78 (0.63–0.92)	69.0	81.0	65.4	0.72

Abbreviations: AUROC, area under the curve; PDI, performance developmental index; MDI, mental developmental index. Model 1 used only MR volumetry data, Model 2 used clinical features exclusively, and Model 3 was developed using both MR volumetry and clinical features.
